# Neutrophils Discriminate between Lipopolysaccharides of Different Bacterial Sources and Selectively Release Neutrophil Extracellular Traps

**DOI:** 10.3389/fimmu.2016.00484

**Published:** 2016-11-04

**Authors:** Elmar Pieterse, Nils Rother, Cansu Yanginlar, Luuk B. Hilbrands, Johan van der Vlag

**Affiliations:** ^1^Department of Nephrology, Radboud University Medical Center, Nijmegen, Netherlands

**Keywords:** NETosis, neutrophil extracellular traps, lipopolysaccharides, platelets, cell death

## Abstract

The release of neutrophil extracellular traps (NETs), either during “suicidal” or “vital” NETosis, represents an important strategy of neutrophils to combat Gram-negative bacteria. Lipopolysaccharide (LPS), a major component of the outer membrane of Gram-negative bacteria, is a reported stimulus for NET formation. Although it is widely acknowledged that the structural diversity in LPS structures can elicit heterogeneous immune responses, species- and serotype-specific differences in the capacity of LPS to trigger NET formation have not yet been investigated. In the present study, we compared the NET-inducing potential of LPS derived from *Escherichia coli* (serotypes *O55:B5, O127:B8, O128:B12, O111:B4*, and *O26:B6*), *Salmonella enterica* (serotype *enteritidis*), and *Pseudomonas aeruginosa* (serotype *10*), under platelet-free and platelet-rich conditions *in vitro*, and in whole blood *ex vivo*. Here, we demonstrate that under serum- and platelet-free conditions, mimicking tissue circumstances, neutrophils discriminate between LPS of different bacterial sources and selectively release NETs only in response to LPS derived from *E. coli O128:B12* and *P. aeruginosa 10*, which both induced “suicidal” NETosis in an autophagy- and reactive oxygen species (ROS)-dependent, but TLR4-independent manner. Intriguingly, in whole blood cultures *ex vivo*, or *in vitro* in the presence of platelets, all LPS serotypes induced “vital” NET formation. This platelet-dependent release of NETs occurred rapidly without neutrophil cell death and was independent from ROS formation and autophagy but required platelet TLR4 and CD62P-dependent platelet–neutrophil interactions. Taken together, our data reveal a complex interplay between neutrophils and LPS, which can induce both “suicidal” and “vital” NETosis, depending on the bacterial origin of LPS and the presence or absence of platelets. Our findings suggest that LPS sensing by neutrophils may be a critical determinant for restricting NET release to certain Gram-negative bacteria only, which in turn may be crucial for minimizing unnecessary NET-associated immunopathology.

## Introduction

Neutrophils are the most abundant terminally differentiated leukocytes circulating in the blood. Attracted by a chemotactic gradient of chemokines, neutrophils can rapidly traffic to inflammatory sites, where they utilize their antimicrobial arsenal of effector mechanisms to eradicate pathogens. In addition to phagocytosis and degranulation, the release of neutrophil extracellular traps (NETs) represents a key antimicrobial strategy of neutrophils (1). NETs are released during a highly complex cell death pathway known as “suicidal” NETosis and comprise an expelled web of chromatin fibers that can bind pathogens, thereby inhibiting their spreading and facilitating their elimination (2, 3). Recent evidence indicates that NETs can also be released from viable neutrophils during an alternative pathway called “vital” NETosis, which requires activated platelets and is therefore thought to occur predominantly during sepsis (4, 5).

The release of NETs has been observed in response to many different bacteria, viruses, fungi, and parasites (6). Nevertheless, it is still incompletely understood how pathogens induce signaling events that result in NETosis. For Gram-negative bacteria, lipopolysaccharide (LPS) has been reported as important stimulus for NETosis (1). However, seemingly contradicting data in literature question whether the interaction between LPS and neutrophils can indeed trigger NET formation. Some reports describe that LPS often protects neutrophils against apoptosis but fails to induce NETosis (7, 8), whereas other reports claim that LPS-induced NETosis is only observed when additional factors are present, such as the immunomodulatory GM-CSF (9), apoptotic microparticles (10), or platelets (11).

Lipopolysaccharide has three main structural components: lipid A, a core domain containing an oligosaccharide component and a repetitive glycan polymer referred to as the O-antigen (12). Whereas the lipid A structure is relatively conserved, there is great variability in the composition of the O-antigen between bacterial strains, which provides the major basis for bacterial serotyping. The structural diversity in LPS has been associated with heterogeneous immune responses (13–16). However, species- and serotype-specific differences in the capacity of LPS to trigger NET formation have not yet been investigated. This study was undertaken to investigate the hypothesis that neutrophils are able to discriminate between LPS structures and thereby selectively release NETs in response to certain structures, which could partly explain the seemingly contradicting data concerning LPS-induced NETosis. Here, we compared the NET-inducing potential of commercially available LPS derived from seven different bacterial sources, i.e., *Escherichia coli* (serotypes *O55:B5, O127:B8, O128:B12, O111:B4*, and *O26:B6*), *Salmonella enterica* (serotype *enteritidis*), and *Pseudomonas aeruginosa* (serotype *10*), under serum- and platelet-free or platelet-rich conditions, mimicking tissue and blood circumstances, respectively.

## Materials and Methods

### Antibodies, Proteins, and Chemicals

Reagents were obtained from the following manufacturers: Sytox Orange (ThermoFisher Scientific, Cat. No. S11368, Duisburg, Germany), *N*-Methoxysuccinyl-Ala-Ala-Pro-Val p-nitroanilide (Sigma-Aldrich, Cat. No. M4765, Schnelldorf, Germany), phorbol 12-myristate 13-acetate (PMA; Sigma-Aldrich, Cat. No. P8139, Schnelldorf, Germany), micrococcal nuclease (MNase; Worthington Biochemical Corporation, Cat. No. LS004798, Lakewood, USA), TNF-α (eBioscience, Cat. No. 14-8329, Frankfurt, Germany), IL-6 (Prospec, Cat. No. cyt-213, Rehovot, Israel), IFN-α (Prospec, Cat. No. cyt-520, Rehovot, Israel), wortmannin (Enzo Life Sciences, Cat. No. BML-ST415, Raamsdonksveer, The Netherlands), diphenyleneiodonium chloride (DPI; Enzo Life Sciences, Cat. No. BML-CN240, Raamsdonksveer, The Netherlands), PKH26 Red Fluorescent Cell Linker Kit (Sigma-Aldrich, Cat. No. PKH26GL, Schnelldorf, Germany), polyclonal anti-TLR4 (InvivoGen, Cat. No. pab-hstlr4, Toulouse, France), anti-myeloperoxidase (BioLegend, Cat. No. 812801, Uithoorn, The Netherlands), anti-neutrophil elastase (Abcam, Cat. No. ab21595, Cambridge, UK), and anti-CD62P (Santa Cruz, Cat. No. sc-8419, Heidelberg, Germany). All LPS structures used in this study were purchased from Sigma-Aldrich (Schnelldorf, Germany) and are listed in Table 1.

**Table 1:** **LPS structures used in this study**.

Species	Serotype	Abbreviation	Cat. No.
*Escherichia coli*	*O55:B5*	LPS-O55	L6529
	*O127:B8*	LPS-O127	L4516
	*O128:B12*	LPS-O128	L2755
	*O111:B4*	LPS-O111	L4391
	*O26:B6*	LPS-O26	L2654
*Salmonella enterica*	*Enteritidis*	LPS-SE	L7770
*Pseudomonas aeruginosa*	*10*	LPS-PA	L9143

### Isolation of Neutrophils

Neutrophils were isolated as described earlier (17). Briefly, neutrophils were isolated at room temperature from EDTA-anticoagulated whole blood by Ficoll density gradient centrifugation using Lymphoprep™ (Stemcell Technologies, Cat. No. 07851). After centrifugation for 20 min at 800 × *g*, the lower cellular fraction with neutrophils was collected, and residual erythrocytes were lysed in a hypotonic buffer. Neutrophils were counted with CASY cell counting technology (Scharfe System, Reutlingen, Germany) and adjusted to 1 million cells per milliliter in serum-free DMEM/F12 medium containing no phenol red (Life Technologies, Cat. No. 11039-021, Bleiswijk, The Netherlands).

### Isolation of Platelets

After Ficoll density gradient centrifugation, platelet-rich plasma was collected and diluted 10 times in a buffer of PBS, 1% FCS, and 1 mM EDTA. Remaining leukocytes were pelleted at 190 × *g* for 15 min at room temperature, after which the remaining supernatant with platelets was pelleted at 2500 × *g* for 5 min at room temperature. The platelet pellet was immediately and carefully resuspended in DMEM/F12 medium to an equivalent volume as they were in the blood, yielding a solution of 100% platelets (v/v).

### Induction and Quantification of NETosis

Purified neutrophils (3 × 10^5^ cells per cm^2^) were seeded in well plates and stimulated with LPS from different bacterial sources, at the indicated concentrations and conditions, for 3–5 h at 37°C. Where indicated, stimulation of neutrophils with 100 nM PMA served as a positive control. After stimulation, neutrophils and adherent NETs were carefully washed twice with pre-warmed PBS (37°C) and isolated by partial NET digestion in DMEM/F12 medium supplemented with 5 U/ml MNase (20 min at 37°C). Extracellular DNA in NET-containing supernatants was stained with 100 nM Sytox Orange and quantified by fluorometry (excitation/emission 530/640 nm). The activity of NET-associated neutrophil elastase (NE) and myeloperoxidase (MPO) was determined colorimetrically, using 100 μM *N*-methoxysuccinyl-Ala-Ala-Pro-Val 4-nitroanilide (at 405 nm) or 1 mM 3,3′,5,5′-tetramethylbenzidine (at 605 nm) as substrates for NE and MPO, respectively.

### Immunofluorescence Imaging

Purified neutrophils (3 × 10^5^ cells per cm^2^) were seeded in slideflask chambers (Thermo Scientific, Cat. No. 170920, Duisburg, Germany) and stimulated with LPS from different bacterial sources, at the indicated concentrations and conditions, for 3–5 h at 37°C. Where indicated, stimulation of neutrophils with 100 nM PMA served as a positive control. After stimulation, cells and NETs were fixed in 4% paraformaldehyde (30 min, room temperature), and slides were stained for DNA (Sytox Orange; 100 nM), NE (dilution 1:200; antibody listed above), and/or MPO (dilution 1:100; antibody listed above). Slides were embedded in Vectashield Mounting Medium (Brunschwig Chemie, Cat. No. H-1200, Amsterdam, The Netherlands), and pictures were obtained with a Zeiss fluorescence microscope with Axiovision software (Sliedrecht, The Netherlands).

### Statistical Analyses

Values are expressed as mean ± SEM. Significance was either determined by Student’s *t*-test or one-way ANOVA followed by Bonferroni correction using GraphPad Prism 5.0 (La Jolla, CA, USA). *p* values less than 0.05 were considered as statistically significant.

## Results

### Neutrophils Selectively Release NETs in Response to Different LPS Structures

**Figure 1:**
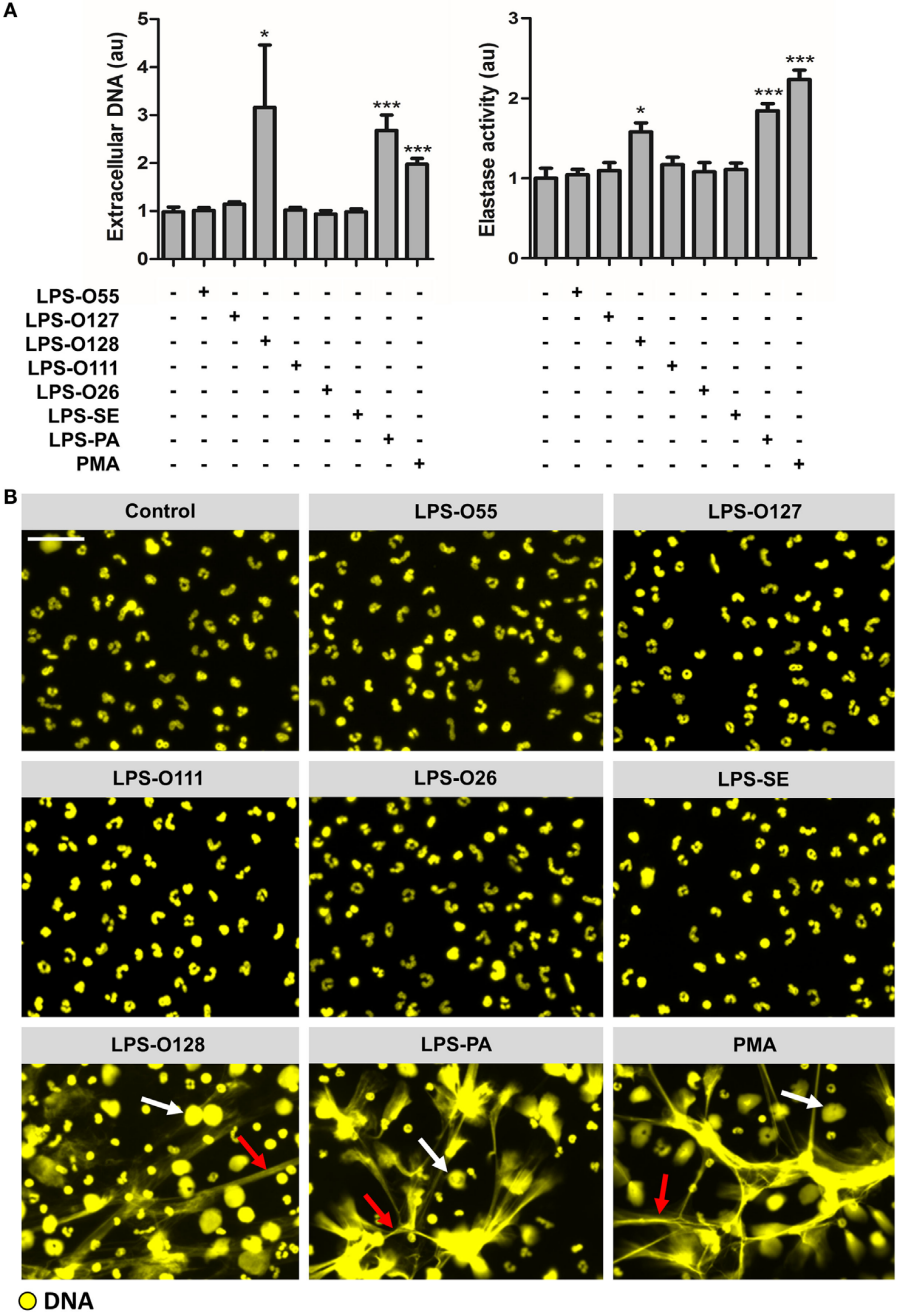
**Neutrophils selectively release NETs in response to LPS structures**. **(A)** LPS-O128 and LPS-PA stimulate neutrophils to release NETs after 180 min of incubation in platelet-free cell cultures (left panel), as measured by fluorometry. The release of DNA by LPS-O128 and LPS-PA coincides with extracellular activity of neutrophil elastase (right panel). **(B)** NET release in response to LPS-O128 and LPS-PA was confirmed by immunofluorescence microscopy, in which DNA was stained with 100 nM Sytox Orange (yellow). Both “spread” NETs (red arrows) and “diffused” NETs (white arrows) were observed. NET release in response to 100 nM PMA served as positive control. LPS was used at a concentration of 8 pg LPS per neutrophil. Scale bar: 30 μm. *p < 0.05 and ***p < 0.001, when compared to control. Data represent mean values ± SEM of at least three experiments.

To evaluate whether LPS is capable of inducing NETosis, and whether there are species- and/or serotype-specific differences in the capacity of LPS to induce NETosis, purified neutrophils were exposed to seven different LPS structures (at a concentration of 8 pg LPS per neutrophil, which is equivalent to ~10 μg/ml LPS) under platelet- and serum-free conditions (thereby largely approaching tissue circumstances), after which NETosis was quantified by measuring DNA release. Extracellular DNA was only detected when neutrophils were exposed to LPS-O128 and LPS-PA, whereas the other LPS serotypes did not induce DNA release (Figure 1A, left panel). For LPS-O128 and LPS-PA, the amount of extracellular released DNA approached ~40–50% of the total cellular DNA, as determined in total cell lysates, indicating that approximately half of the neutrophils were lysed and released NETs. Measurement of extracellular elastase activity in the same culture supernatants revealed that only LPS-O128 and LPS-PA were able to induce the release of elastase (Figure 1A, right panel). The release of both extracellular DNA and elastase clearly suggested that NETs were released. Indeed, the presence of NETs could be confirmed by immunofluorescence imaging, where typical DNA filaments (“spread” NETs) as well as “diffused” NETs could be observed (Figure 1B). In sum, LPS-induced NETosis is under tissue circumstances species- and serotype-dependent and is, among the seven LPS structures investigated here, limited to LPS-O128 and LPS-PA.

### LPS-O128 and LPS-PA Induce NETosis Only When Present above a Threshold Value

**Figure 2:**
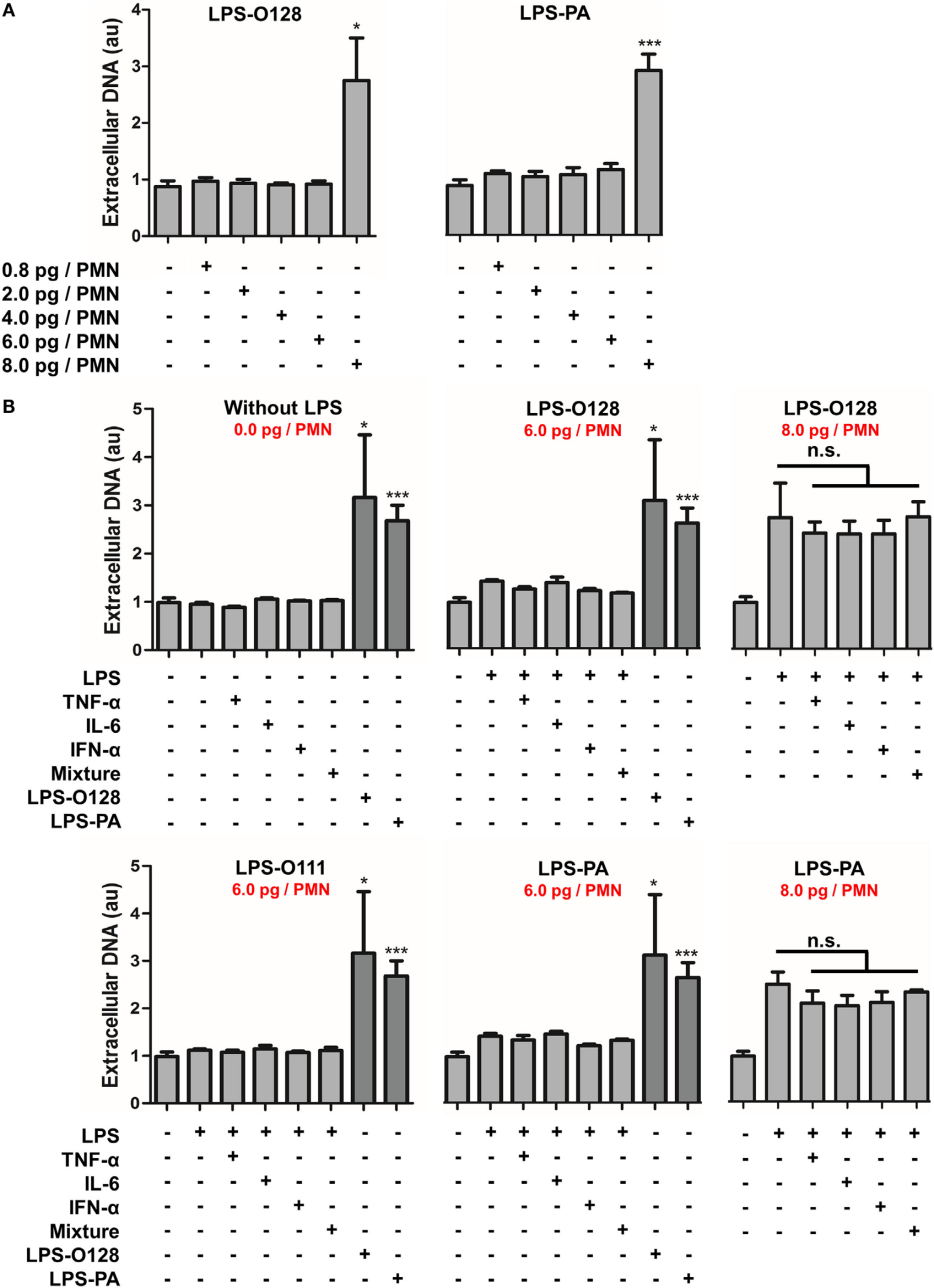
**LPS-O128 and LPS-PA induce NETosis when present above a threshold value**. **(A)** LPS-O128 and LPS-PA induce NET release only at high LPS concentrations of 8 pg LPS per neutrophil (PMN). **(B)** Priming of neutrophils for 1 h with recombinant TNF-α (10 ng/ml), IL-6 (10 ng/ml), IFN-α (100 ng/ml), or a mixture of all, does not promote NET release nor prime neutrophils for LPS-induced NETosis by LPS-O111 (bottom left), LPS-O128 (top middle), and LPS-PA (bottom middle) at a concentration of 6 pg LPS per neutrophil (PMN). In these graphs, NETosis induced by LPS-O128 and LPS-PA at 8 pg LPS per neutrophil is shown in dark as positive controls. Cytokines do not enhance NETosis by LPS-O128 or LPS-PA at LPS concentrations of 8 pg LPS per neutrophil (top and bottom right). Quantifications of NET release were performed by fluorometry, as outlined. *p < 0.05, **p < 0.01, and ***p < 0.001, when compared to control. Data represent mean values ± SEM of at least three experiments.

Next, the NET-inducing capacity of LPS-O128 and LPS-PA was tested at lower concentrations under the same serum- and platelet-free circumstances. Intriguingly, lowering LPS concentrations did not result in a gradual decrease of the number of neutrophils undergoing NETosis, but instead all neutrophils remained unaffected below concentrations of 8 pg of LPS-O128 and LPS-PA per neutrophil (Figure 2A). This indicates that a certain minimum quantity of LPS-O128 and LPS-PA is required to exceed a threshold value that induces NETosis in neutrophils. It can be hypothesized that a pro-inflammatory milieu, i.e., the presence of pro-inflammatory cytokines, might lower the threshold for the induction of NETosis by LPS-O128 and LPS-PA. To test this, neutrophils were preincubated for 1 h with the cytokines TNF-α (10 ng/ml), IL-6 (10 ng/ml), or IFN-α (100 ng/ml), or a mixture of these cytokines, after which LPS was added at a concentration just below the threshold (6 pg LPS per neutrophil). The pro-inflammatory cytokines neither induced NETosis themselves (Figure 2B, top left panel) nor lowered the threshold for NETosis induced by LPS-O128 and LPS-PA (Figure 2B, middle panels). In addition, LPS-O111 did not gain NET-inducing capacity by priming neutrophils with pro-inflammatory cytokines (Figure 2B, bottom left panel). Also, the pro-inflammatory cytokines did not enhance NETosis by LPS-O128 and LPS-PA at a concentration of 8 pg LPS per neutrophil (Figure 2B, right panels). In summary, LPS-O128 and LPS-PA trigger NETosis when they are present above a certain threshold value, which is independent from a pro-inflammatory milieu.

### LPS-O128 and LPS-PA Induce ROS- and Autophagy-Dependent “Suicidal” NETosis

**Figure 3:**
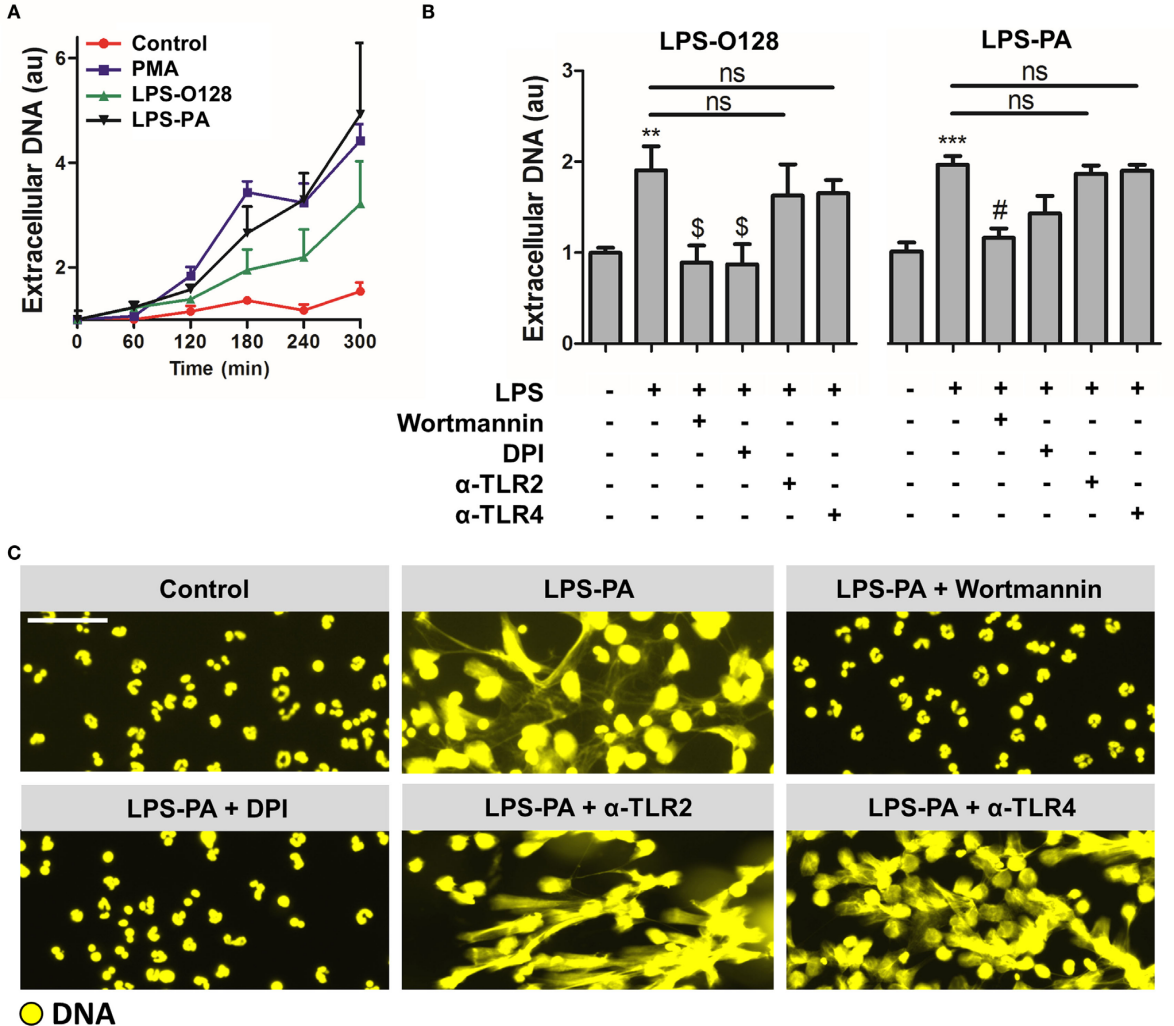
**LPS-PA and LPS-O128 induce ROS- and autophagy-dependent “suicidal” NETosis**. **(A)** DNA release in response to 100 nM PMA, LPS-O128, or LPS-PA was monitored by fluorometry using 100 nM Sytox Orange during an incubation period of 5 h. **(B)** NETosis induced by LPS-O128 and LPS-PA can be inhibited by 5 μM wortmannin (inhibitor of autophagy) or 40 μM diphenyleneiodonium (DPI; inhibitor of ROS), but not by anti-TLR2 and anti-TLR4 neutralizing antibodies (5 μg/ml). **(C)** The inhibitory effects of wortmannin and DPI on NET release by LPS-PA were confirmed by immunofluorescence microscopy, in which DNA was stained with 100 nM Sytox Orange (yellow). Immunofluorescence imaging also confirmed that anti-TLR2 and anti-TLR4 neutralizing antibodies did not prevent NETosis induced by LPS-PA. LPS was used at a concentration of 8 pg LPS per neutrophil. Scale bar: 30 μm. **p < 0.01 and ***p < 0.001, when compared to control, ^$^*p* < 0.05 and ^#^*p* < 0.01, when compared to LPS-PA alone. Data represent mean values ± SEM of at least three experiments.

As outlined, NETs can be generated in several ways, i.e., through cell death-associated “suicidal” NETosis or through “vital” NETosis (5). Since the viability of neutrophils stimulated with LPS-O128 and LPS-PA under serum- and platelet-free conditions (i.e., tissue circumstances) appeared heavily altered (Figure 1B), as witnessed by altered lobulated nuclei and decondensed chromatin, we tested the hypothesis that LPS-O128 and LPS-PA trigger the canonical cell death-associated pathway of “suicidal” NETosis, which is dependent on reactive oxygen species (ROS) and autophagy and usually takes hours to complete (7). Indeed, LPS-induced NETosis typically occurred after 3 h (Figure 3A) and could be fully prevented by inhibition of autophagy (using wortmannin) or ROS [using diphenyleneiodonium (DPI)] (Figures 3B,C). Since LPS signaling is heavily dependent on TLR4, and arguably to a lesser extent on TLR2, we assessed whether blockade of TLR2 and TLR4 could prevent NET release in response to LPS-O128 and LPS-PA. However, inhibition of TLR2 and TLR4 did not alter NET release in response to either LPS structure, suggesting that LPS-induced NETosis occurs in a TLR4 and TLR2-independent manner (Figure 3B). Collectively, these data indicate that LPS-PA and LPS-O128 induce “suicidal” ROS- and autophagy-dependent NETosis under tissue circumstances, which does not require TLR2 or TLR4.

### LPS Induces “Vital” NETosis in the Presence of Platelets

**Figure 4:**
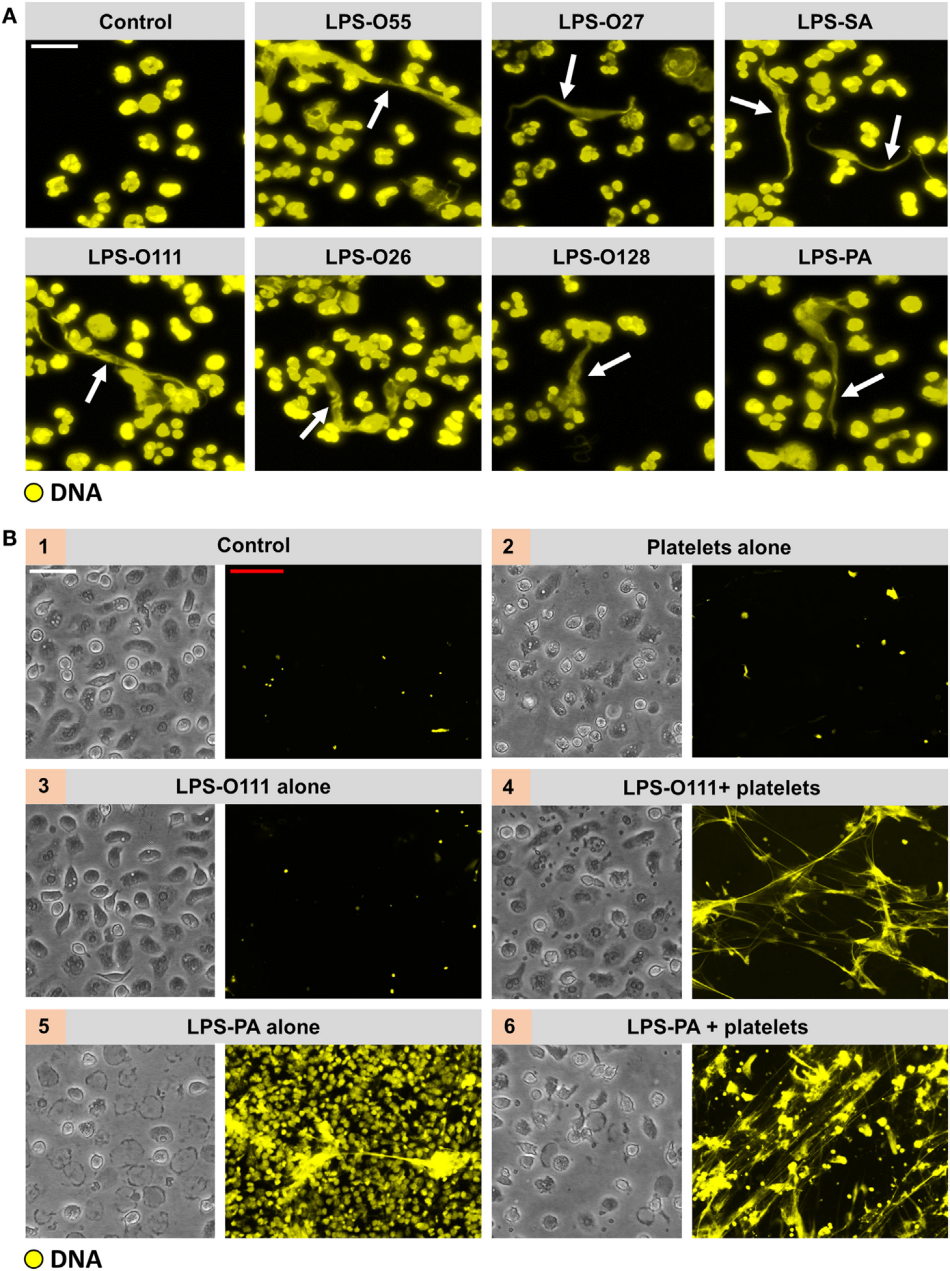
**All LPS structures induce “vital” NETosis in the presence of platelets**. **(A)** Typical extracellular DNA filaments (white arrows) were observed in whole blood cultures *ex vivo* after 180 min of incubation with the different LPS serotypes. **(B)** Platelets (panel 2) or LPS-O111 (panel 3) alone does not induce NETosis after 180 min of incubation with neutrophils, whereas the combination of both (panel 4) stimulates NET formation without neutrophil (lytic) cell death. Massive neutrophil cell death is observed in response to LPS-PA alone (panel 5), based on the failure to exclude the vital dye Sytox Orange (yellow), which can be largely prevented by the addition of platelets (panel 6). LPS was used at a concentration of 8 pg LPS per neutrophil. Notably, representative light microscopy images are shown to visualize neutrophil morphology after stimulation and do not correspond in terms of “field of view” to the adjacent representative immunofluorescence images. Scale bars: white = 20 μm and red = 40 μm.

**Figure 5:**
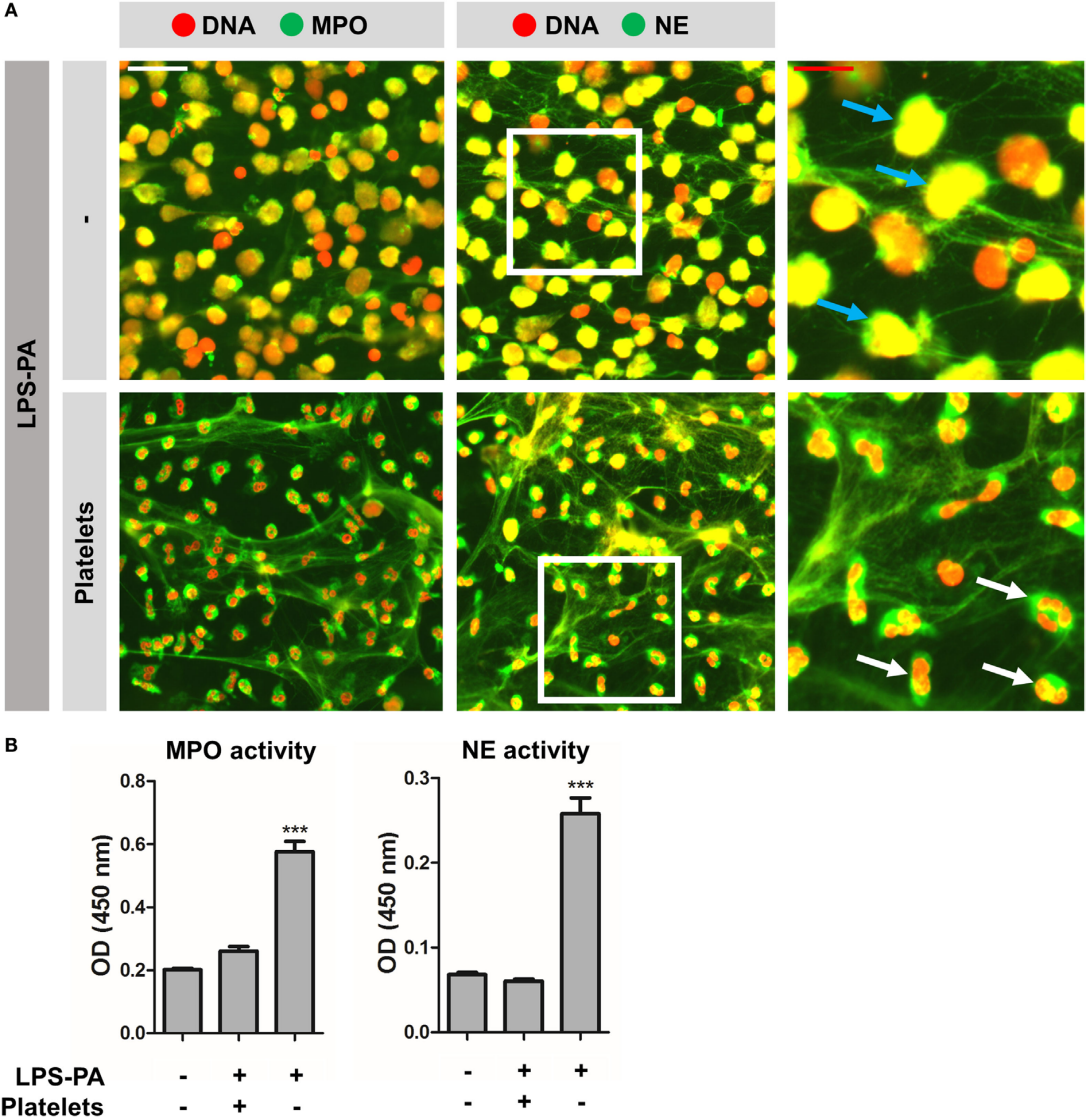
**“Vital” NETs lack proteolytic active myeloperoxidase and elastase**. **(A)** “Vital” NETs induced by platelets exposed to LPS-PA, as well as “suicidal” NETs induced by LPS-PA alone, stain positive for both myeloperoxidase (MPO) and neutrophil elastase (NE). Note the highly refined architecture of thinly interwoven DNA filaments of “vital” NETs when compared to “suicidal” NETs. Also note (right panels, inserts) the granular and intact neutrophil phenotype (i.e., lobulated nuclei) for “vital” NETs (white arrows) when compared to the altered neutrophil phenotype (i.e., decondensed chromatin) for “suicidal” NETs (blue arrows). **(B)** “Vital” NETs induced by platelets exposed to LPS-PA lack proteolytic active myeloperoxidase (MPO) and neutrophil elastase (NE) when compared to “suicidal” NETs induced by LPS-PA alone. For these assays, NETs were isolated through digestion with micrococcal nuclease and normalized on the basis of DNA content in NET-containing supernatants. Scale bars: white = 40 μm and red = 20 μm. ***p < 0.001, when compared to control. Data represent mean values ± SEM of at least three experiments.

It was recently demonstrated that LPS-activated platelets induce “vital” NETosis during sepsis (4, 5). This form of NET release is fundamentally different from “suicidal” NETosis; hence, “vital” NETosis occurs much faster, is not dependent on autophagy or ROS, and is not associated with direct lytic cell death. Therefore, the NET-inducing capacity of all seven LPS structures was retested in the presence of platelets. Intriguingly, in whole blood *ex vivo*, thus in the presence of platelets, NET-like DNA lattices could be identified in response to all seven LPS structures (Figure 4A). An *in vitro* coculture setting of neutrophils and isolated platelets indeed revealed NETosis in response to LPS structures that previously failed to induce NETosis under platelet-free conditions, for instance LPS-O111 (Figure 4B). Whereas platelets alone or LPS-O111 failed to trigger NET release, the combination of platelets and LPS-O111 resulted in robust NET formation (Figure 4B, panels 2–4). Platelet-dependent LPS-induced NETs were observed already within 60 min of incubation, and the majority of neutrophils retained the capacity to exclude the vital dye Sytox Orange, which is indicative for “vital” NETosis (Figure 4B, panel 4). On the contrary, neutrophils stimulated with LPS-PA only did not exclude Sytox Orange anymore, which is indicative for “suicidal” NETosis (Figure 4B, panel 5). Importantly, “suicidal” NETosis induced by LPS-PA could be largely prevented by the addition of platelets (Figure 4B, panels 5 and 6). Finally, to confirm that the extracellular DNA fibers in response to LPS-exposed platelets represent “vital” NETs, double stainings for DNA with either MPO or elastase (NE) were performed. Indeed, extracellular DNA co-localized with both MPO and NE when neutrophils were stimulated with platelets exposed to LPS-PA (Figure 5A; single channel images as Figure S1 in Supplementary Material), thereby confirming that the observed DNA structures are “vital” NETs. However, the proteolytic activity of both MPO and NE within these “vital” NETs appeared to be lower when compared to “suicidal” NETs induced by LPS-PA alone (Figure 5B). In conclusion, LPS-exposed platelets mediate “vital” NETosis independent from the bacterial origin of LPS.

### Platelet TLR4 and CD62P Are Required for LPS-Induced “Vital” NETosis

**Figure 6:**
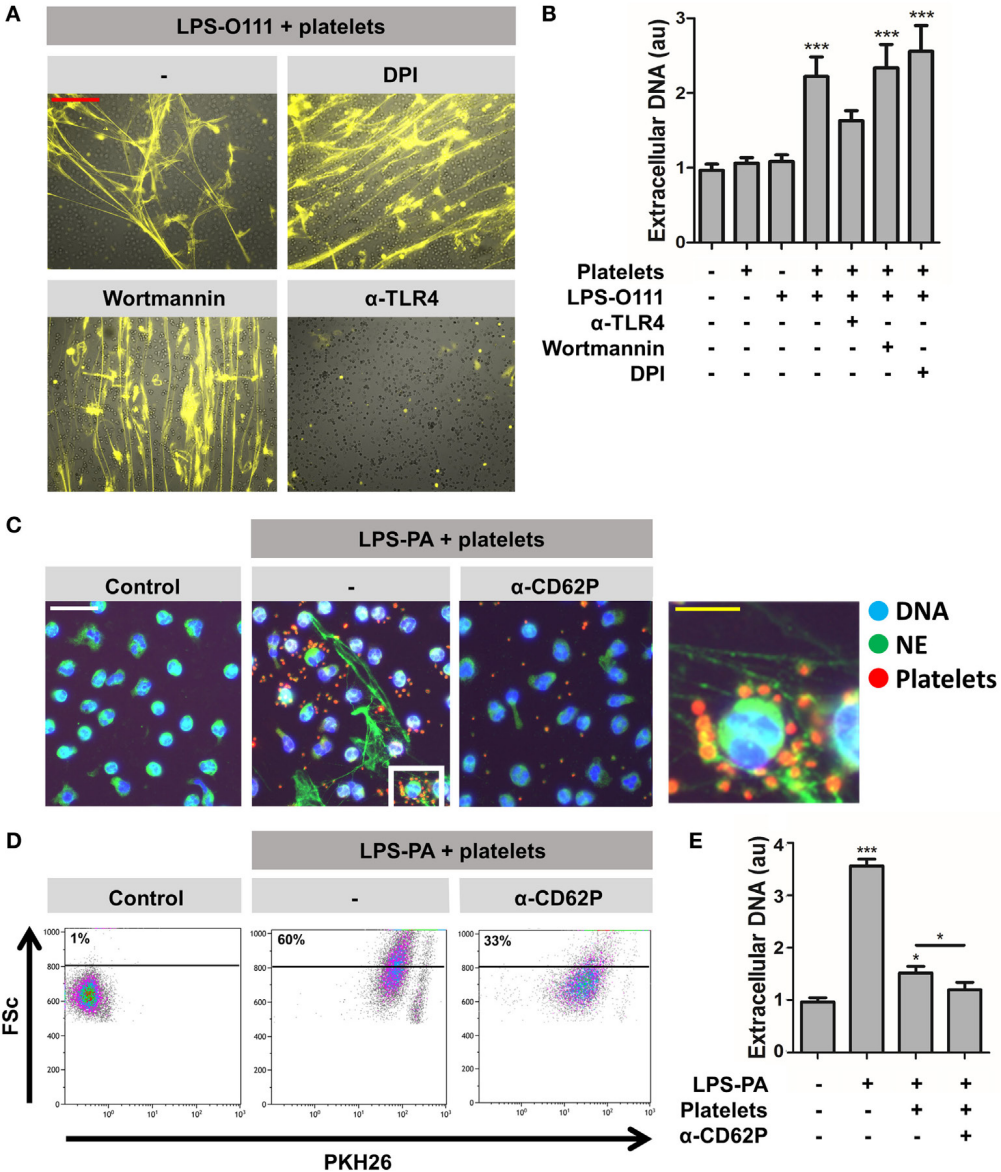
**Platelet-mediated “vital” NETosis requires platelet TLR4 and CD62P**. **(A,B)** “Vital” NETosis induced by LPS-O111 and platelets is insensitive to inhibition by 5 μM wortmannin (inhibitor of autophagy) or 40 μM diphenyleneiodonium (DPI; inhibitor of ROS) but can be prevented by pretreatment of platelets with anti-TLR4 neutralizing antibodies (5 μg/ml). Representative images are merged pictures of extracellular DNA (yellow, as stained with 100 nM Sytox Orange) and neutrophils (brightfield channel). The images show abundant NETs despite exclusion of Sytox Orange by neutrophils, indicating cell death-independent NET release (i.e., “vital” NETosis). Quantification of NET release **(B)** was performed by fluorometry, as outlined. **(C)** PKH26-labeled platelets (red) stimulated with LPS-PA form aggregates with neutrophils (NE, neutrophil elastase) after 30 min of incubation. This aggregate formation is inhibited by anti-CD62P-neutralizing antibodies. **(D)** Flow cytometry analysis confirms the formation of platelet–neutrophil aggregates, since cells within the predefined neutrophil gate increase in size (FSc, forward scatter) and stain positive for PKH26-labeled platelets after LPS-PA stimulation. **(E)** Anti-CD62P-neutralizing antibodies decrease platelet-mediated “vital” NETosis induced by LPS-PA, as measured by fluorometry. LPS was used at a concentration of 8 pg LPS per neutrophil. Scale bars: red = 100 μm; white = 30 μm; yellow = 10 μm. *p < 0.05 and ***p < 0.001, when compared to control, where not indicated. Data represent mean values ± SEM of at least three experiments.

The mechanisms involved in platelet-dependent LPS-induced “vital” NETosis may involve platelet TLR4, which was previously shown to be required for “vital” NETosis (11). Indeed, anti-TLR4 neutralizing antibodies could inhibit NET release in response to LPS-O111 to a large extent (Figures 6A,B). The inhibition of ROS (with DPI) or autophagy (with wortmannin) did not influence “vital” NETosis induced by LPS-O111 (Figures 6A,B; single channel images as Figure S2 in Supplementary Material). Besides promoting “vital” NETosis, platelets apparently also exerted inhibitory effects on “suicidal” NETosis induced by LPS-PA (Figure 4B, panels 5 and 6). In the presence of platelets, LPS-PA hardly promoted cell death, while NETs remained present to the same extent as for neutrophils exposed to LPS-PA in the absence of platelets. Thus, platelets seem to be important mediators in directing the neutrophil’s fate during NET release, whereas LPS-PA induces “vital” NETosis in the presence of platelets, “suicidal” NETosis is induced when platelets are absent. As an explanation for this observation, it can be hypothesized that LPS-exposed platelets are internalized by neutrophils, thereby delivering NE to phagosomes and sequestering NE from the nucleus, which in turn inhibits “suicidal” NETosis since nuclear elastase is required in this pathway (18, 19). To test this hypothesis, PKH26-labeled platelets exposed to LPS-PA were cocultured with neutrophils, after which PKH26-labeled platelets and NE were visualized by immunofluorescence imaging. Apparently, platelets were not internalized by neutrophils, but instead formed large aggregates with neutrophils (Figure 6C, middle panel; single channel images as Figure S3 in Supplementary Material). The platelet–neutrophil aggregates coincided with the presence of NETs, consisting of extracellular filaments of DNA and NE (Figure 6C, insert right panel). The formation of platelet–neutrophil aggregates was further analyzed by flow cytometry, which revealed an increased forward scatter and PKH26-positivity in the neutrophil population (Figure 6D). Since CD62P is crucial for platelet–neutrophil interactions (20), PKH26-labeled platelets exposed to LPS-PA were cocultured with neutrophils in the presence of CD62P-neutralizing antibodies. Indeed, CD62P-neutralizing antibodies could largely prevent the formation of LPS-PA-induced platelet–neutrophil aggregates (Figures 6C,D) and decreased NET release (Figure 6E). Collectively, these data demonstrate a dominant role for platelets in LPS-induced “vital” NETosis, which occurs in an autophagy- and ROS-independent, but platelet TLR4- and platelet CD62P-dependent manner.

## Discussion

This study shows that neutrophils are able to discriminate between LPS of different bacterial sources and thereby selectively release NETs. Under serum- and platelet-free conditions, thereby mimicking tissue circumstances, neutrophils released NETs in response to two out of seven tested different LPS structures, of which LPS derived from *P. aeruginosa* (LPS-PA) appeared particularly potent. *P. aeruginosa* is a pathogen that typically infects the respiratory and urinary tract, as well as chronic wounds, and is a significant cause of morbidity and mortality in hospitalized patients (21). It is well-accepted that neutrophils comprise a pivotal component of host protection against *P. aeruginosa* (22). It was recently reported that *P. aeruginosa* is a robust instigator of NET formation *in vitro* and *in vivo* within the lungs, which contribute to the pathogenesis of airway changes in patients with bronchiectasis and cystic fibrosis (23–26). It remains unelucidated why LPS-PA is particularly potent in eliciting NET release when compared to the LPS derived from other bacterial species. Since *P. aeruginosa* is generally perceived as a non-virulent opportunist, it is unclear why this microorganism would elicit an antimicrobial defense mechanism (i.e., NETosis) that is associated with collateral tissue damage (6), rather than triggering conventional phagocytosis. Reasoning otherwise, the potent NET-inducing capacity of LPS-PA could explain why this bacterium is actually perceived as an opportunist; hence, NETosis represents an extremely powerful strategy of constraining bacterial traits (27). An explanation for the NET-inducing capacity of LPS-PA may lie in the fact that *P. aeruginosa* is well-known for its ways to circumvent phagocytosis, for example through the formation of biofilms (28). Thus, NETosis may provide an immune response to those bacteria that have evolved strategies to circumvent phagocytic killing. Indeed, multiple other pathogens notorious for their attempts to evade phagocytosis, such as *Streptococcus pneumonia* (29), *Haemophilus influenza* (30), and *Klebsiella pneumoniae* (31), are potent stimuli for NETosis. Thus, phagocytosis and NETosis may have a complementary role, whereby the failure to phagocytose may elicit NET release. In line with this is the observation that NETosis is triggered by fungi that are too large for phagocytosis, whereas small hyphae become phagocytosed without inducing NETosis (18).

In addition to LPS-PA, LPS from *E. coli* (serotype *O128:B12*; LPS-O128) induced NETosis under tissue circumstances. Of note, four other tested LPS serotypes of *E. coli* (serotypes *O55:B5, O127:B8, O111:B4*, and *O26:B6*) did not elicit NET release. Thus, LPS-induced NETosis is not only bacterial species-specific but also serotype-specific. Growing evidence supports the notion that inflammatory responses triggered by LPS vary among serotypes. For instance, in a murine model of infection-induced preterm labor, four different *E. coli* LPS serotypes yielded highly variable outcomes (32). Our data suggest that subtle changes in the sugar composition of the O-antigen can impact NET release in response to LPS. It has been shown that modulation of the O-antigen composition alters the recognition and consecutive phagocytosis of LPS molecules by macrophages (33), which may also hold for neutrophils. In line with the reciprocal relationship between NETosis and phagocytosis described above, NETosis may thus be triggered by certain O-antigens that facilitate bypassing of phagocytosis.

Notably, the selectivity of NET release in response to LPS structures in our study was lost when neutrophils were cocultured with platelets. Also, extracellular chromatin fibers typical for NETs could be identified in whole blood *ex vivo* in response to all seven different LPS structures. Thus, in the presence of platelets, there is no selectivity of NET release in response to the different LPS structures. We observed that neutrophil–platelet interactions induced a rapid release of NETs (<60 min) in response to all LPS structures, which occurred in a ROS-independent manner and preceded without evident lysis of neutrophils. This form of rapid NET release could largely be inhibited through the blockade of platelet CD62P. Indeed, it has previously been shown that CD62P promotes NETosis in mice (34). The binding of CD62P, also known as P-selectin, to P-selectin glycoprotein ligand-1 (PSGL-1) on neutrophils thus causes signaling events that result in NETosis. However, the exact molecular mechanisms underlying CD62P-induced NET release remain elusive. Nevertheless, many downstream pathways of PSGL-1, such as the Src/Syk, PI3K/Akt, and p38 MAPK pathways, have in other contexts already been shown to be involved in NET release (35–37).

Our observations that LPS-exposed platelets rapidly induce NET release independent from ROS and independent from neutrophil lysis closely resemble a form of NETosis, which has previously been referred to as “vital” NETosis (5). However, the existence of a non-cell death “vital” NETosis program has been doubted by some critics, who question whether a neutrophil can still live and function without an intact nucleus (38, 39). Nevertheless, there is evidence that anuclear granulocytes are in fact metabolically active and able to perform cellular functions, such as transmigration (40) and phagocytosis (41). Simultaneously, some researchers have been critical toward fundamental aspects of “suicidal” NETosis, who perceive “suicidal” NETosis rather harmful than beneficial for the host due to its robust nature (42). Furthermore, it remains controversial whether “suicidal” NETosis is truly a unique form of cell death or whether it in fact reflects other forms of cell death, such as necroptosis (38, 43). Nonetheless the above, we have adapted the terms “suicidal” and “vital” NETosis in this manuscript, since these terms have been defined in previously published work in which the reported observations correspond to our findings. However, we agree that at least the term “vital” NETosis is a *contradictio in terminis*, since the “osis” of NETosis implies death and “vital” implies alive. Although our data support the coexistence of both “suicidal” and “vital” NETosis, whereby platelets ultimately direct the neutrophil’s fate, further investigation is required to fully understand “vital” and “suicidal” processes and to assess the reciprocal relationship between both. Moreover, there should be international consensus about the terminology applied to describe the different forms of NET release as well as neutrophil cell death.

The structure of “vital” NETs induced by LPS-exposed platelets appeared highly refined and sophisticated, forming much larger structures of thinly interwoven DNA filaments when compared to “suicidal” NETs. Furthermore, we found that peroxidase and elastase activity was lacking in “vital” NETs, in contrast to “suicidal” NETs. These two characteristics of “vital” NETs (i.e., the complex web-like structure and the lack of proteolytic activity) makes “vital” NETs highly suitable for trapping and encapsulating pathogens, but presumably not for direct extracellular killing. However, since neutrophils appear to remain viable during “vital” NETosis, subsequent phagocytosis of entrapped pathogens may follow, and this combination of NET release and phagocytosis may provide an efficient strategy to combat pathogens in the bloodsteam during sepsis without inducing protease-mediated collateral tissue damage to the vessel lumen. Thus, aside from the idea that NETosis may occur when phagocytosis is impaired, there is also evidence that NETosis and phagocytosis by neutrophils can occur simultaneously and complement each other during septic conditions (44).

In addition to the biological relevance, our data may also have technical experimental implications. Since many studies addressing the contribution of NETosis to disease conditions (i.e., systemic lupus erythematosus) depend on the *in vitro* induction and isolation of NETs for downstream assays, the choice for LPS as an inducer of NET release should be selected carefully in light of the current data (6, 45–47).

**Figure 7:**
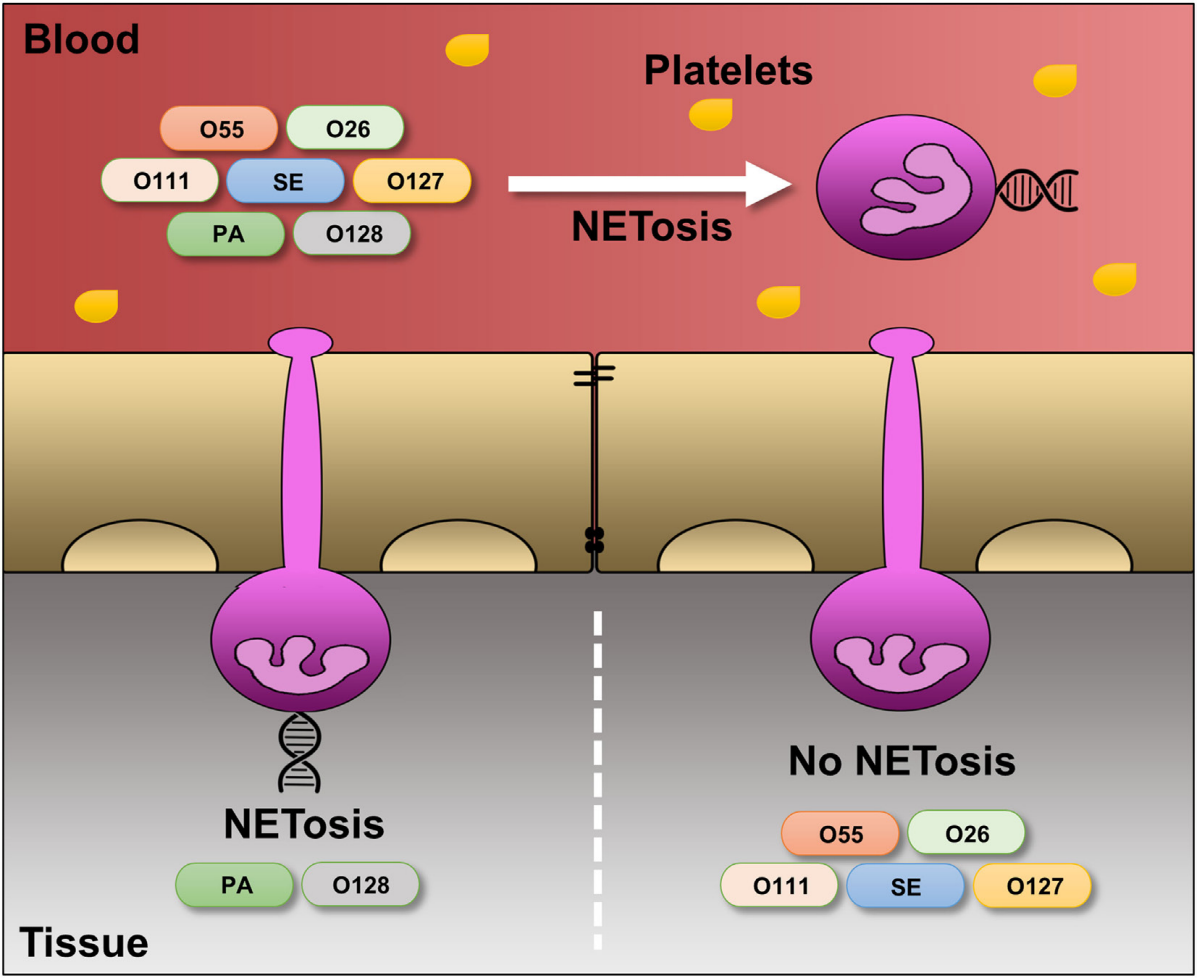
**Differential regulation of LPS-induced NET release under platelet-free and platelet-rich circumstances**. Under serum- and platelet-free conditions, mimicking tissue circumstances, LPS-PA and LPS-O128 trigger ROS- and autophagy-dependent “suicidal” NET release in extravasated neutrophils, whereas other LPS structures (LPS-O26, LPS-O55, LPS-SE, LPS-O127, and LPS-O111) do not. In the presence of platelets, mimicking blood circumstances, neutrophils do no longer discriminate between LPS structures and release “vital” NETs in response to all LPS structures.

Taken together, our data reveal a complex interplay between neutrophils and LPS, which can induce both “suicidal” and/or “vital” NETosis, depending on the quantity and structure of the LPS and the presence or absence of platelets (Figure 7). Although the present study compares the effects of a single molecule (LPS) from different bacterial sources, it is tempting to speculate that similar results may be obtained for whole microbes. This could be addressed in future research.

## Author Contributions

EP designed and performed research, analyzed and interpreted data, and wrote the manuscript; NR and CY performed research and analyzed data; LH interpreted data and wrote the manuscript; and JV designed and supervised research, interpreted data, and wrote the manuscript.

## Conflict of Interest Statement

The authors declare that the research was conducted in the absence of any commercial or financial relationships that could be construed as a potential conflict of interest.
