# Supplementary material for: Neutrophils Discriminate between Lipopolysaccharides of Different Bacterial Sources and Selectively Release Neutrophil Extracellular Traps
Source: Front Immunol. 2016 Nov 4;7:484. doi: 10.3389/fimmu.2016.00484 (PMC5095130; doi:10.3389/fimmu.2016.00484)
Supplement: Supplementary file 1 [file Data_Sheet_1.DOCX]

Neutrophils discriminate between lipopolysaccharides of different bacterial sources and selectively release neutrophil extracellular traps

Elmar Pieterse^1^, Nils Rother^1^, Cansu Yanginlar^1^, Luuk Hilbrands^1^ and Johan van der Vlag^1*^

^1^Department of Nephrology, Radboud University Medical Center, Nijmegen, the Netherlands

*** Correspondence:**J. van der Vlag, Nephrology Research Laboratory (480), Department of Nephrology, Radboud University Medical Center, Geert Grooteplein 10, 6525 GA Nijmegen, The Netherlands, Tel: +31-24-3616539, E-mail: [johan.vandervlag@radboudumc.nl](mailto:johan.vandervlag@radboudumc.nl)

Keywords: NETosis, neutrophil extracellular traps, lipopolysaccharides, platelets, cell death

Supplementary Data

**
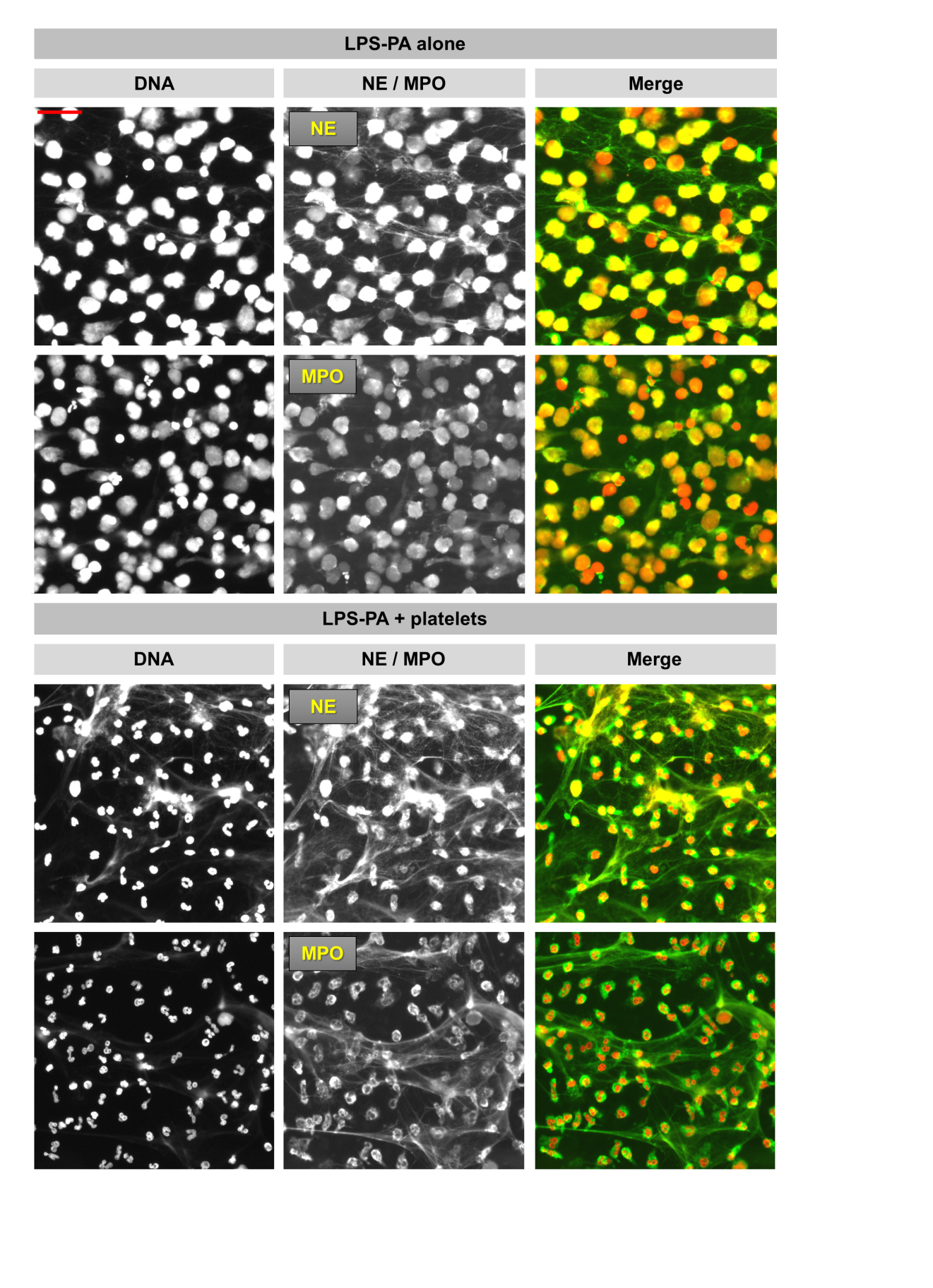
**

**Supplementary Figure 1 – Single channel fluorescence images of Figure 5a.** ‘Vital’ NETs induced by platelets exposed to LPS-PA, as well as ‘suicidal’ NETs induced by LPS-PA alone, stain positive for both myeloperoxidase (MPO) and neutrophil elastase (NE). Note the highly refined architecture of interwoven DNA filaments of ‘vital’ NETs when compared to ‘suicidal’ NETs. Also note (right panels, inserts) the granular and intact neutrophil phenotype for ‘vital’ NETs when compared to the altered neutrophil phenotype for ‘suicidal’ NETs. *Scale bar: 40 µm.*

**
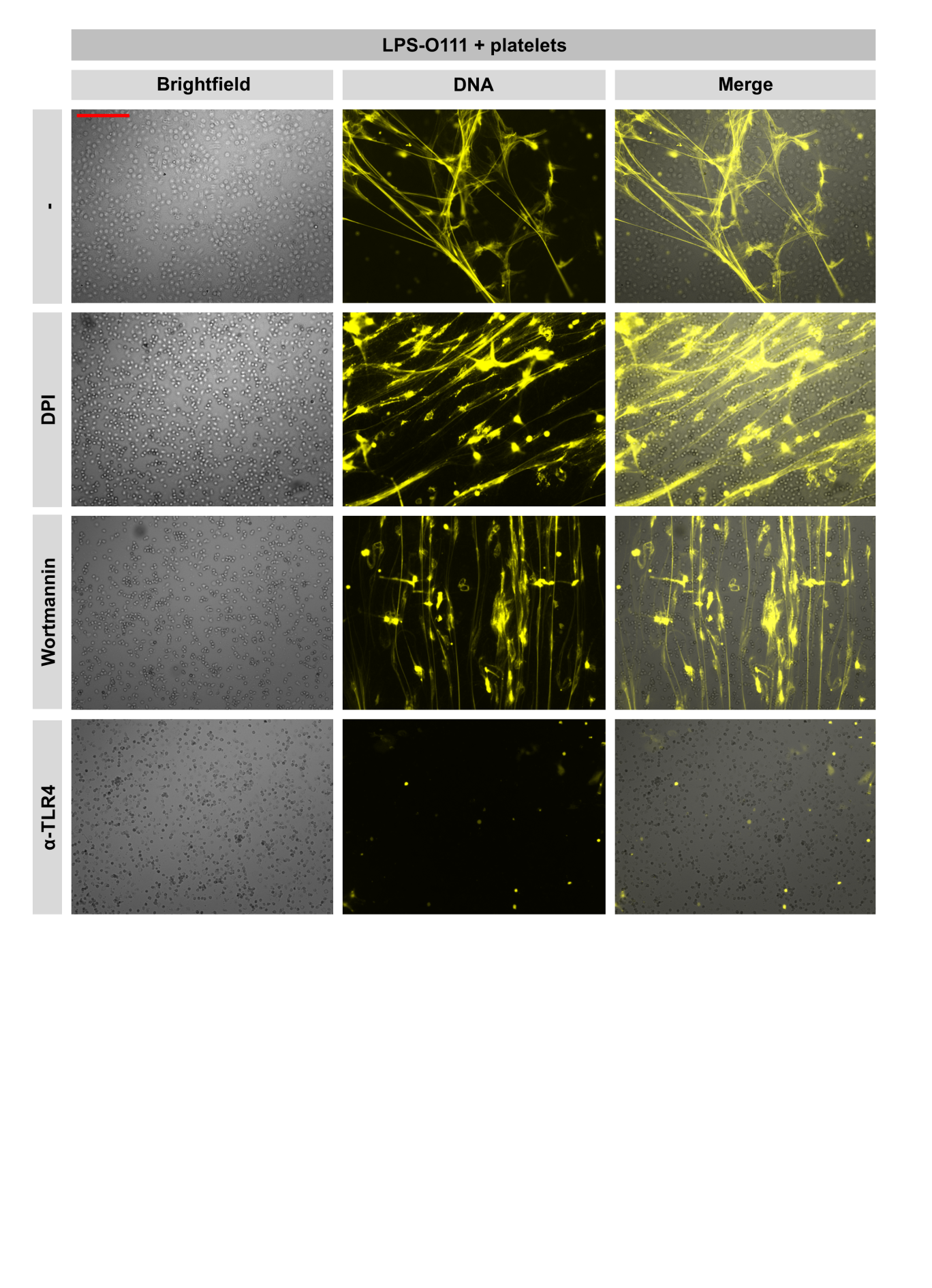
**

**Supplementary Figure 2 – Single channel fluorescence images of Figure 6a.** ‘Vital’ NETosis induced by LPS-O111 and platelets is insensitive to inhibition by 5 µM wortmannin (inhibitor of autophagy) or 40 µM diphenyleneiodonium (DPI; inhibitor of ROS), but can be prevented by pre-treatment of platelets with anti-TLR4 neutralizing antibodies (5 µg/ml). Representative images are merged pictures of extracellular DNA (yellow, as stained with 100 nM Sytox Orange) and neutrophils (brightfield channel). The images show abundant NETs despite exclusion of Sytox Orange by neutrophils, indicating cell death-independent NET release, i.e. ‘vital’ NETosis. *Scale bar: 100 µm.*

**
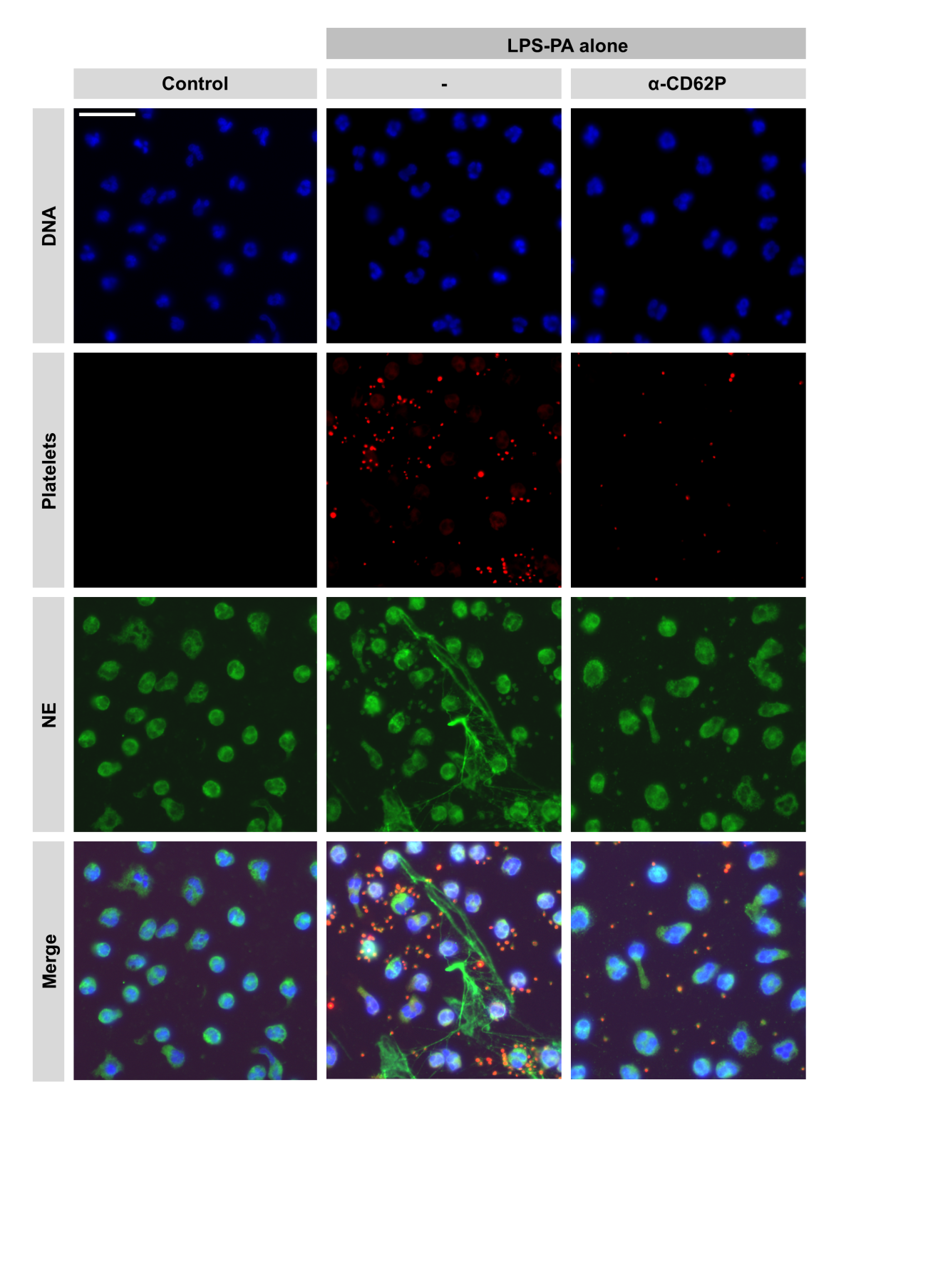
**

**Supplementary Figure 3 – Single channel fluorescence images of Figure 6c.** PKH26-labeled platelets (red) stimulated with LPS-PA form aggregates with neutrophils (NE; neutrophil elastase) after 30 min. of incubation. This aggregate formation is inhibited by anti-CD62P neutralizing antibodies. *Scale bar: 30 µm.*
